# Caloric restriction is associated with preservation of muscle strength in experimental cancer cachexia

**DOI:** 10.18632/aging.101724

**Published:** 2018-12-26

**Authors:** Stef Levolger, Sandra van den Engel, Gisela Ambagtsheer, Jan N.M. IJzermans, Ron W.F. de Bruin

**Affiliations:** 1 Department of Surgery, Erasmus MC – University Medical Center, Rotterdam, The Netherlands

**Keywords:** caloric restriction, cachexia, muscle wasting, cancer

## Abstract

Caloric restriction increases lifespan and healthspan, and limits age-associated muscle wasting. In this study, we investigate the impact of 30% caloric restriction (CR) in a murine cancer cachexia model. Forty CD2F1 mice were allocated as C26 tumor-bearing (TB) + ad libitum food intake (dietary reference intake [DRI]), TB CR, non-TB (NTB) CR, or NTB matched intake (MI). TB groups were inoculated subcutaneously with 0.5x10^6^ C26 cells 14 days after initiating CR. Bodyweight, food intake, and grip-strength were recorded periodically. Gastrocnemius (GCM) and tibialis anterior (TA) muscles were resected and weighed 3 weeks after tumor inoculation. mRNA expression of MuRF1, Atrogin-1, myogenin, and MyoD was determined. At tumor inoculation, the mean body weight of TB CR was 88.6% of initial body weight and remained stable until sacrifice. TB DRI showed wasting before sacrifice. TB groups experienced muscle wasting compared with NTB MI. Grip-strength change was less severe in TB CR. Expression of MuRF1, Atrogin-1, and MyoD was similar between TB DRI and both CR groups. Expression of myogenin was increased in CR groups. In conclusion, caloric restriction limits loss of muscle strength but has no impact on muscle mass despite significant loss of body weight in an experimental cancer-associated cachexia model.

## Introduction

Cancer cachexia describes a syndrome of progressive weight loss due to muscle wasting with or without the loss of adipose tissue, anorexia, and abnormal metabolism in the presence of underlying cancer [1]. It cannot be reversed by conventional nutritional support and leads to progressive functional impairment [1,2]. Nearly half of all cancer patients are faced with cachexia in the course of their disease, and it is the cause of death in up to 20 percent [3-5]. Catabolic cytokines and patient-related factors such as age are key pathogenic mechanisms underlying cancer cachexia [6-8]. Catabolic pro-inflammatory cytokines associated with cancer cachexia include interleukin-6 (IL-6), interleukin-1 beta (IL-1B), tumor necrosis factor alpha (TNF-α), and interferon gamma (IFN-γ) [7,9].

Particularly IL-6 is found highly upregulated in the final months preceding death [10]. Treatment aimed at reducing the synthesis of pro-inflammatory cytokines or blocking their action may, therefore, contribute to improved physical performance and quality of life [11-13].

Besides novel pharmaceutical strategies to limit the activity of catabolic cytokines in cancer cachexia, dietary interventions have sparked great interest [11,13-19]. Thus far dietary interventions for the treatment of cancer cachexia have evaluated supplementation therapy. Long-chain omega-3 fatty acid eicosapentaenoic acid (EPA) is one of the most frequently investigated supplements. Systematic reviews of the literature published since have been unable to support clinical application of EPA for the treatment of cancer cachexia [18,20]. Only smaller studies initially reported to limit weight loss in cancer patients [21]. In contrast to this, β-hydroxy-β-methylbutyrate (HMB), a leucine metabolite, and quercetin have been found to limit experimental muscle wasting in vivo [14-16] as well as in a clinical trial following a 24-week supplementation program [22]. Similarly, another study found a strong trend towards the preservation of muscle mass in advanced cancer patients following 8 weeks of HMB supplementation [23]. Although counterintuitive, caloric restriction (CR) may elicit similar effects. The beneficial effects of CR on healthspan and longevity have been thoroughly established in model organisms, and include reduced incidence of cancer, cardiovascular disease, and increased oxidative stress resistance [24-31]. Experiments in our own laboratory have shown that two weeks of 30% CR improves insulin sensitivity, increased insulin/insulin-like growth factor 1 signaling, increases expression of markers of antioxidant defense, and reduces expression of markers of inflammation in mice [29]. In rodents and nonhuman primates, CR was able to limit sarcopenia, i.e. the age-related loss of muscle mass [32-35]. Similarly as in cancer cachexia, catabolic pro-inflammatory cytokines are suggested to play an important role in the development of sarcopenia [36,37].

Therefore, we questioned whether CR could limit muscle wasting and loss of muscle function in an experimental cancer cachexia model and we examined the impact of CR on body weight, muscle weight, and grip-strength. In addition, the mRNA expression levels of skeletal muscle catabolic E3 ubiquitin ligases and anabolic myogenic regulatory factors were studied.

## RESULTS

To study the effects of 30% caloric restriction forty male CD2F1 mice were allocated to four groups. Mice allocated to be C26 tumor-bearing (TB) animals with ad libitum access to chow were used as dietary reference intake (DRI) for all other mice in this experiment, i.e. C26 TB mice on a 30% caloric restriction (CR) diet; non-tumor bearing (NTB) mice with matched intake to the TB-DRI group (MI); NTB mice on a 30% caloric restriction diet.

**Figure 1:**
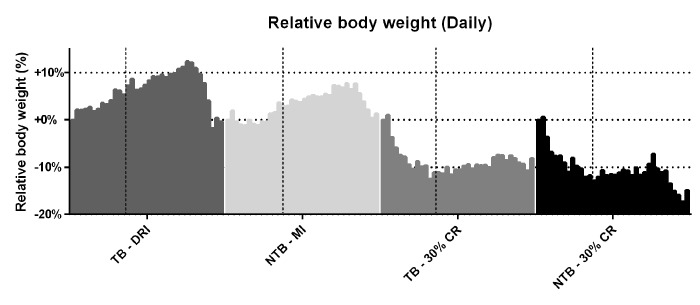
**Daily body weight throughout the experiment.** Grouped histograms depicting the mean daily bodyweights per group in C26 tumor-bearing (TB) male CD2F1 mice with ad libitum access to chow (dietary reference intake [DRI], n = 10); C26 TB mice on a 30% caloric restriction (CR, n = 10) diet; non-tumor bearing (NTB) mice with matched intake (MI, n = 10); NTB mice on a 30% caloric restriction (n = 10). The vertical dashed lines indicate the timepoint in the experiment in which tumor inoculation was performed in tumor-bearing groups. The vertical bars indicate daily measurements of body weight, ranging from day 0 to 35, for each specified group. Bodyweight was normalized to each animal’s body weight on day 0 and is expressed as the percental difference. Following initiation of 30% CR a rapid decline in body weight was observed prior to tumor inoculation, -10.5% for C26 TB 30% CR mice and -10.6% for NTB 30% CR mice (p < 0.001 for both groups compared to C26 TB DRI). Following tumor inoculation, C26 TB DRI mice experienced a 10.6% drop in bodyweight preceding sacrifice (p = 0.01, paired-sample *t*-test), whereas C26 TB 30% CR mice had a steady bodyweight in this phase of the experiment. NTB MI mice experienced a 6.4% drop in body weight (p = 0.002, paired-sample *t*-test) and NTB 30% CR mice experienced a 7.6% drop in body weight (p = 0.004, paired-sample *t*-test) preceding sacrifice.

**Figure 2:**
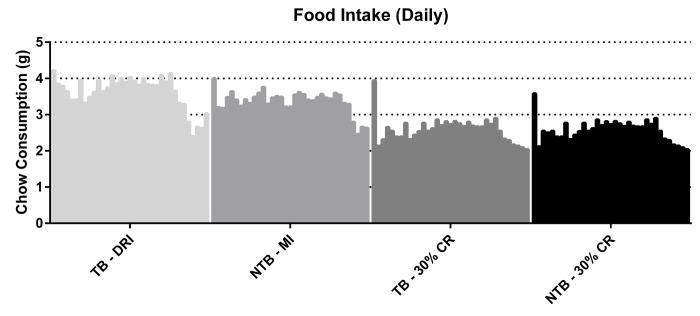
**Daily food intake throughout the experiment.** Grouped histograms depicting the mean daily food intake per group in C26 tumor-bearing (TB) male CD2F1 mice with ad libitum access to chow (dietary reference intake [DRI], n = 10); C26 TB mice on a 30% caloric restriction (CR, n = 10) diet; non-tumor bearing (NTB) mice with matched intake (MI, n= 10); NTB mice on a 30% caloric restriction (n = 10). The vertical bars indicate daily measurements of food intake, ranging from day 0 to 35, for each specified group. Food intake is expressed as grams (g). Food intake of C26 TB DRI mice decreased in the final days preceding sacrifice from 3.8 g to 2.9 g (p = 0.0002, paired-sample *t*-test). Consequently, food intake decreased in the other groups accordingly.

Following initiation of 30% CR, a rapid but similar decline in body weight was observed in both CR groups (NTB-CR and TB 30% CR) (Figure 1). This loss of bodyweight was most apparent in the first week, prior to inoculation of the C26 adenocarcinoma cells. Consequently, this loss of bodyweight was attributable to 30% CR alone. Mice allocated to the NTB MI group had access to an equal amount of food per cage as consumed the day prior by C26 TB DRI mice. Despite this, the NTBI MI group consumed significantly less than the TB DRI mice during the first 7 days of the experiment, i.e. prior to actual tumor inoculation (Figure 2). The mean intake of C26 TB DRI mice was 3.7 g versus 3.4 g in NTB MI mice (p = 0.03). This difference in food intake between the NTB MI and C26 TB DRI groups was associated with a lower maximum increase in bodyweight. At tumor inoculation, mean body weight in TB 30% CR mice was 88.6% of initial body weight compared with 106.9% in the TB DRI mice. Following tumor inoculation, mice in the TB DRI group gained bodyweight until 28 days after the start of the experiment. From day 28 until sacrifice at day 35, animals lost 10.6% of initial body weight (p = 0.01). This was associated with a decrease in food intake from 3.8 g to 2.9 g (p = 0.0002). Consequently, NTB MI mice too experienced a loss of 6.4% in body weight in these final days of the experiment (p=0.002). Mice in the TB 30% CR group had a stable bodyweight following tumor inoculation, and no further decrease in body weight was observed (p = 0.186). Mice in the NTB 30% CR group lost 7.6% in mean body weight in the final days of the experiment (p = 0.004). This difference may, in part but not exclusively, be attributed to tumor weight increase in the TB 30% CR group.

**Figure 3:**
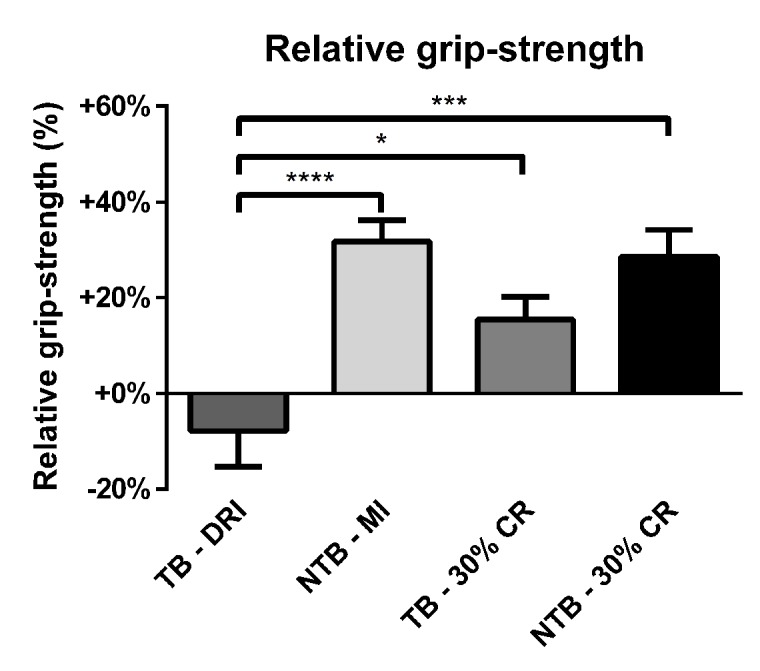
**Relative grip-strength at the end of the experiment.** Bar graphs depicting the mean ± SEM for final grip-strength normalized to starting grip-strength in C26 tumor-bearing (TB) male CD2F1 mice with ad libitum access to chow (dietary reference intake [DRI], n = 10); C26 TB mice on a 30% caloric restriction (CR, n = 10) diet; non-tumor bearing (NTB) mice with matched intake (MI, n = 10); NTB mice on a 30% caloric restriction (n = 10). Multiple group comparisons were done by one-way ANOVA with a Bonferroni’s post hoc test. All groups were compared against TB – DRI mice. Asterisk brackets are displayed for significant results only. * p < 0.05 ** p < 0.01 *** p < 0.001.

A reduction in grip-strength was observed throughout the follow-up period for TB DRI mice (Figure 3). The final mean loss of grip-strength was 7.9% when compared to starting grip-strength. TB 30% CR mice, on the other hand, experienced an increase of 15.4% in grip-strength throughout the experiment. This difference was significant in comparison to the TB DRI mice (p = 0.02). NTB mice, both NTB MI and NTB 30% CR, experienced the greatest increase in grip-strength, which was 31.7% (p < 0.001) and 28.6% (p = 0.0002) respectively at the end of the experiment.

**Figure 4:**
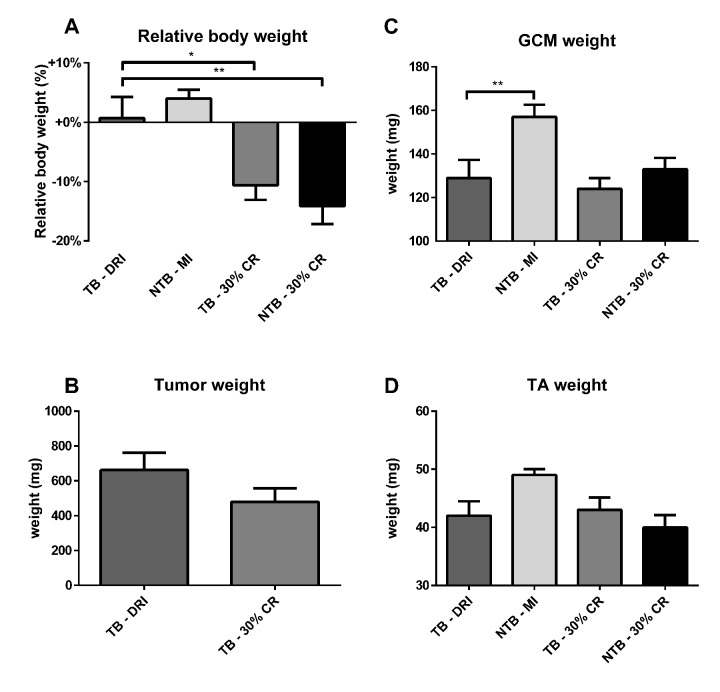
**Body weight, muscle weight and tumor mass at sacrifice.** Bar graphs depicting the mean ± SEM for (**A**) final bodyweight normalized to starting bodyweight, (**B**) tumor weight, (**C**) gastrocnemius muscle weight and (**D**) tibialis anterior muscle weight in C26 tumor-bearing (TB) male CD2F1 mice with ad libitum access to chow (dietary reference intake [DRI], n = 10); C26 TB mice on a 30% caloric restriction (CR, n = 10) diet; non-tumor bearing (NTB) mice with matched intake (MI, n = 10); NTB mice on a 30% caloric restriction (n = 10). Multiple group comparisons were done by one-way ANOVA with a Bonferroni’s post hoc test. All groups were compared against TB – DRI mice. Asterisk brackets are displayed for significant results only. * p < 0.05 ** p < 0.01 *** p < 0.001. Statistical comparison between TB DRI and TB 30% CR mice in tumor weight was done by Student’s *t*-test (p = 0.17).

All animals were sacrificed at 21 days following tumor inoculation, i.e. 35 days after onset of the experiment. At sacrifice, the final decrease in bodyweight was greatest in TB 30% CR and NTB 30% CR mice, 10.5% and 14.0% respectively (Figure 4A). As expected, NTB MI mice had an increase in body weight of 4.0%. TB DRI mice experienced a rapid decline in body weight in the final days preceding sacrifice by 10.6%. Tumor mass increased until day 21, when resected mean tumor weight was 662 ± 316 mg in TB DRI mice versus 480 ± 249 mg in TB 30% CR mice. This trend towards reduced tumor growth in CR mice was not significant (p = 0.17) (Figure 4B). Furthermore, no association between tumor weight and body weight loss was observed. Directly following sacrifice, the gastrocnemius and tibialis anterior muscles were resected and weighed. Mean gastrocnemius muscle weight in NTB MI mice was 158.3 ± 18.3 mg versus 128.7 ± 25.3 mg in TB DRI mice, p = 0.008 (Figure 4C). Mean gastrocnemius muscle weight for C26 TB 30% CR mice was 124.4 ± 15.5 mg, comparable to C26 TB DRI mice (p > 0.99). Similarly, mean gastrocnemius muscle weights for NTB 30% CR mice were 132.5 ± 15.4 mg, comparable to C26 TB DRI mice (p > 0.99). Mean tibialis anterior muscle weight in NTB MI mice was 48.9 ± 3.4 mg versus 42.1 ± 8.5 mg in the C26 TB DRI mice (p = 0.08) (Figure 4D). Mean tibialis anterior muscle weights for C26 TB 30% CR mice were 42.6 ± 5.4 mg, comparable to C26 TB DRI mice (p > 0.99). Similarly, mean tibialis anterior muscle weights for NTB 30% CR mice were 40.0 ± 4.9 mg, comparable to C26 TB DRI mice (p > 0.99).

**Figure 5:**
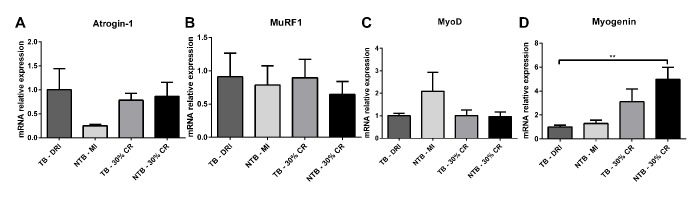
**mRNA expression levels in cachectic muscle.** Bar graphs depicting the mean ± SEM mRNA expression levels in gastrocnemius muscle of (**A**) Atrogin-1, (**B**) MuRF1, (**C**) MyoD and (**D**) Myogenin in C26 tumor-bearing (TB) male CD2F1 mice with ad libitum access to chow (dietary reference intake [DRI], n = 10); C26 TB mice on a 30% caloric restriction (CR, n = 10) diet; non-tumor bearing (NTB) mice with matched intake (MI, n = 10); NTB mice on a 30% caloric restriction (n = 10). Multiple group comparisons were done by one-way ANOVA with a Bonferroni’s post hoc test. All groups were compared against TB – DRI mice. Asterisk brackets are displayed for significant results only. * p < 0.05 ** p < 0.01 *** p < 0.001.

Skeletal muscle E3 ubiquitin ligases and myogenic regulatory factors mRNA expression profiles were determined in gastrocnemius muscle samples. A substantial, non-significant difference in E3 ubiquitin ligase atrogin-1 expression was observed between C26 TB DRI and NTB MI (Figure 5A). No difference was observed between C26 TB DRI, C26 TB 30% CR and NTB 30% CR. Expression of the second E3 ubiquitin ligase MuRF1 and myogenic regulatory factor MyoD were comparable between all four groups (Figure 5B, 5C). Finally, and perhaps most interesting, there was increased expression of the myogenic regulatory factor myogenin in the NTB 30% CR group (p = 0.002) as well as a substantial, non-significant elevation in the TB 30% CR group (Figure 5D).

## DISCUSSION

Cancer-associated cachexia is a common finding in patients affected by numerous types of malignancies [38,39]. Unfortunately, there are still no treatment modalities to halt or reverse this process of muscle wasting. Previously it was shown that caloric restriction may decrease age-related sarcopenia [32-34]. Our study investigated whether caloric restriction might protect against muscle wasting and loss of muscle function. Although counterintuitive, our findings show that in the C26 cancer cachexia model, caloric restriction had no impact on muscle wasting when compared to ad libitum fed TB mice. Moreover, the mRNA expression of E3 ubiquitin ligases MuRF1 and Atrogin-1 expression was unaffected by 30% caloric restriction. This suggests a protective mechanism by which CR prevents aggravated muscle wasting. This was also reflected in grip-strength. The final grip-strength in the TB 30% CR group was greater than the final grip-strength in TB DRI mice. Nonetheless, this grip-strength was still decreased compared to both NTB MI as well as NTB 30% CR mice. CR alone had no impact on grip strength in non-tumor-bearing mice. Similar findings have been previously reported [40]. Discrepancies between muscle mass and muscle strength have also been noted in human populations [41-43]. Taken together, these findings show a limited protective effect on the functional outcome of CR in tumor-bearing mice, which is not powerful enough to prevent loss of muscle strength. This protective effect may be attributed to the enhanced expression of myogenin in mice on a 30% caloric restriction diet. Similar effects of myogenin have previously been described following myogenin gene transfer in an ALS model [44]. In that study, myogenin gene transfer lead to increased rotarod performance, whilst the bodyweight loss profile remained unaffected.

In addition, mice allocated to receive CR, both tumor-bearing and non-tumor bearing, showed enhanced activity throughout the experiment, e.g. increased running and climbing, as well as being found frequently hanging from the top of the cage. Although we did not quantify these findings, similar results have been reported in an age-related sarcopenia caloric restriction rodent model [40].The increased activity of animals on CR may have contributed to the preservation of grip-strength as well as to myogenin upregulation.

Furthermore, non-tumor-bearing mice on caloric restriction demonstrated a higher mean body weight loss than tumor-bearing on caloric restriction. This difference may in part, but not exclusively, be attributed to tumor weight. Increased organ weight, i.e. liver and spleen, has been reported in C26-bearing mice [45,46] and is likely to have contributed to these difference in body weight. Moreover, considering fluid intake was not monitored a possible contribution of water weight is unknown. Lastly, despite energy intake being fixed, energy expenditure is not. Possible differences in physical activity may too have contributed to these differences.

Studies employing caloric restriction have been primarily aimed at investigating its role in improving the efficacy of anti-cancer therapies [47-50], protecting against anti-cancer therapy side-effects [51,52], as well as preventing oncogenesis [48]. Although the difference in tumor mass was non-significant between the C26 TB DRI and C26 TB 30% CR mice in the current study, an earlier meta-analysis has shown that caloric restriction may reduce tumor growth [53]. This anti-cancer effect has also been described after short-term fasting and fasting cycles [54]. Even though in the current study we did not seek to investigate the anti-cancer effects of caloric restriction, the observed trend towards reduced tumor growth can be regarded as an additional benefit of caloric restriction.

Several limitations apply to the present study. The study was powered on an expected reduction in loss of muscle weight. As such, non-significant differences in secondary outcome parameters (e.g. relative mRNA expression levels) may have been subject to type II errors. Furthermore, survival was not included as one of the endpoints due to the strict ethical guidelines associated with the initiation of this study. Another important consideration is timing of caloric restriction. For this study mice were put on a calorie restricted diet prior to inoculation of cancer cells. This may limit direct translation of these findings to clinical patients, who have established cancer, and may already suffer from anorexia Moreover, a recent study by Boldrin et al. reports that changes induced by caloric restriction in an age-related sarcopenia model do not persist with time, and, perhaps even more important, are dependent on mouse strain and gender differences [55]. Taking our own findings into account we concur with the authors of the aforementioned study to be cautious in applying caloric restriction to improve skeletal muscle function in humans.

In conclusion, we found that caloric restriction limits the loss of muscle strength in vivo in an experimental cancer-associated cachexia model. Caloric restriction did not aggravate the loss of cachexia associated muscle mass despite significant body weight loss. These findings suggest that although caloric restriction does not fully protect against the detrimental effects of cancer-associated cachexia, it does limit muscle strength loss. This suggests that caloric restriction might be safely utilized in improving the efficacy of-, and protect against the adverse side effects of anti-cancer therapies. Further research is warranted to confirm these findings upon initiation of caloric restriction in early and late-stage cancer.

## MATERIALS AND METHODS

### Animal Ethics Committee approval

All animal experiments were performed with the approval of the local Animal Ethics Committee and in accordance with the Dutch National Experiments on Animal Act and complied with the EU adopted Directive 86/609/EEC (1986).

### Animals

Male CD2F1 (BALB/c × DBA/2 F1) mice of 8 weeks weighing approximately 25 grams were purchased from Charles River, Maastricht, the Netherlands. All mice were housed in individually ventilated cages under standard conditions with a 12 h light-dark cycle (n = 3 – 4 animals per cage). Animals were acclimatized for one week prior to the start of the experiments.

### Diet

All animals had ad libitum access to water and CRM (P) chow (Special Diet Services, Witham, Essex, UK) during the acclimatization period and throughout the full duration of the experiment. At the start of the experiment dietary intake was determined in 3 cages that were randomly allocated as to become tumor-bearing (TB). Twenty-four-hour food consumption in these cages was determined daily by weighing the remnant chow and calculating the difference from the preceding day. This was set as the dietary reference intake (DRI) [56]. The other cages were randomly allocated as TB, 30% CR animals (i.e. chow weighing 70% of the DRI); non-tumor bearing (NTB), 30% CR animals; and NTB, matched intake animals (i.e. chow weighing 100% of the DRI of the TB animals). All groups consisted of 10 mice. We did not include an AL-NTB group to control for weight loss due to reduced food intake in the TB mice. The pair fed non-tumor bearing control group we used compensates for the effects of possible reduced food intake by the tumor bearing animals which allows us to discriminate between the effects of reduced food intake per se, and the effects of the combination of the presence of a tumor and reduced food intake.

### Cancer cachexia model

Colon-26 (C26) adenocarcinoma cells were kindly provided by Dr. D.O. McCarthy (Ohio State University, Columbus, OH, USA). These cells were cultured in RPMI 1640 (Westburg BV, Leusden, The Netherlands) supplemented with 10% fetal bovine serum (FBS, Sigma-Aldrich, St. Louis, The United States of America), and 1% penicillin/streptomycin (P/S, Fisher Scientific, Waltham, The United States of America) at 37^o^C with 5% CO_2_. Animals allocated in TB groups received a subcutaneous inoculation in the right flank with 0.5 x 10^6^ C26 adenocarcinoma cells in 100 μL sterile PBS on the 14^th^ day of the experiment. The inoculation was done under anesthesia by isoflurane inhalation (5% isoflurane induction). This is a well-established model of cancer cachexia in mice [57].

### Grip strength assessment

Combined hind- and forelimb grip strength was measured twice per week by placing the animal on a grid attached to a force gauge (BIOSEB, Chaville, France), and steadily pulling the mice by the tail along the sensor axle until grip is released. The maximum strength produced before releasing the grid was registered in triplicate with one minute rest period for each animal. Obtained values were averaged to provide a mean force measurement for each individual animal and subsequently normalized to each animal’s grip-strength respectively on day zero.

### Body weight, muscle mass, and tumor size

Body weight was recorded daily. Tumor size was recorded every other day starting on day 23 of the experiment, i.e. day 9 after tumor inoculation, using digital calipers. Tumor mass was estimated via the formula mass (mg) = tumor volume (mm^3^) = width^2^ x length/2 [58]. Animals were sacrificed by cardiac puncture followed by cervical dislocation under isoflurane anesthesia on day 35 of the experiment, i.e. 21 days after tumor inoculation. Immediately following sacrifice the gastrocnemius (GCM), and tibialis anterior (TA) muscles of both hind legs and tumor were dissected, weighed and immediately snap-frozen in liquid nitrogen and stored at -80 °C until analysis.

### RNA isolation and Real-time polymerase chain reaction

For gene expression analysis, total RNA was isolated from snap-frozen GCM muscle tissue using Trizol reagent (Invitrogen, Breda, the Netherlands), and subsequently purified by DNase treatment (RQ1 RNase-Free DNase) (Promega Benelux B.V., Leiden, the Netherlands). 1 μg of total RNA was reversed transcribed to cDNA using random hexamer primers (Invitrogen, Breda, the Netherlands), and Superscript II RT (Invitrogen, Breda, the Netherlands). Quantitative real-time polymerase chain reaction (RT-PCR) was performed using an iCycler real-time PCR system (Biorad, California, The United States of America) using SYBR Green (Sigma-Aldrich, St. Louis, The United States of America). Used primer sequences can be found in Table 1. GAPDH was used as housekeeping gene for normalization. Relative gene expression was calculated (2^delta delta CT)/ (average 2^delta delta Ct healthy controls) [59]. Each sample was tested in duplicate.

**Table 1:** Reverse transcription-polymerase chain reaction primer sequences.

Gene	Forward Primer	Reverse Primer	Genbank Accession Number
Atrogin1	5’-GTTTTCAGCAGGCCAAGAAG	5’-TTGCCAGAGAACACGCTATG	AF_441120
MyoD	5’-AAACCCCAATGCGATTTATCAGG	5’-TAAGCTTCATCTTTTGGGCGTGA	NM_010866
Myogenin	5’-CACTCCCTTACGTCCATCGT	5’-CAGGACAGCCCCACTTAAAA	NM_031189
Murf1	5’-AGGTGTCAGCGAAAAGCAGT	5’-CCTCCTTTGTCCTCTTGCTG	NM_009066
GAPDH	5’-ATGCATCCTGCACCACCAACT	5’-CAGTGATGGCATGGACTGTG	NM_008084

### Statistics

Categorical data are expressed as number (percentage) and continuous variables as mean ± SEM (normal distribution, visually assessed and by means of the Shapiro-Wilks test). Body weight and grip-strength were normalized to each animal’s body weight and grip-strength respectively on day 0. Muscle weight from the left hind leg and right hind leg were averaged to provide a mean GCM and TA muscle weight for each animal. Multiple group comparisons were done by one-way ANOVA with a Bonferroni’s post hoc test. For comparison between periodic measurements, the paired-sample *t*-test was used. Statistical comparison between TB DRI and TB 30% CR mice in tumor weight was done by Student’s *t*-test. All analyses were performed using IBM SPSS Statistics for Windows, version 21.0 (IBM Corp., Armonk, NY, USA). A *P* value < 0.05 was considered statistically significant.
